# ENDOCRINOLOGY IN THE TIME OF COVID-19: Management of thyroid nodules and cancer

**DOI:** 10.1530/EJE-20-0269

**Published:** 2020-05-20

**Authors:** Alexis Vrachimis, Ioannis Iakovou, Evanthia Giannoula, Luca Giovanella

**Affiliations:** 1 Department of Nuclear Medicine, German Oncology Center, University Hospital of the European University, Limassol, Cyprus; 2 Academic Department of Nuclear Medicine, University Hospital AHEPA, School of Medicine, Thessaloniki, Greece; 3 Academic Department of Nuclear Medicine, General Hospital Papageorgiou, Thessaloniki, Greece; 4 Clinic for Nuclear Medicine and Competence Centre for Thyroid Diseases, Imaging Institute of Southern Switzerland, Bellinzona, Switzerland; 5 Clinic for Nuclear Medicine, Zurich University Hospital, Zurich, Switzerland

## Abstract

Most patients with thyroid nodules and thyroid cancer (TC) referred for diagnostic work-up and treatment are not considered at higher risk of infection from SARS-CoV-2 compared to the general population. On the other hand, healthcare resources should be spared to the maximum extent possible during a pandemic. Indeed, while thyroid nodules are very common, only a small percentage are cancerous and, in turn, most thyroid cancers are indolent in nature. Accordingly, diagnostic work-up of thyroid nodules, thyroid surgery for either benign or malignant thyroid nodules and radioiodine treatment for differentiated thyroid cancers may be safely postponed during SARS-CoV-2 pandemic. Appropriate patient counselling, however, is mandatory and red flags should be carefully identified prompting immediate evaluation and treatment as appropriate. For these selected cases diagnostic work-up (e.g. ultrasound, scintigraphy, fine-needle aspiration), surgery and radioiodine therapy may proceed despite the threat of SARS-CoV-2 infection and COVID-19, after an individual risk-benefit analysis.

## Introduction

With nearly 4.5 million cases confirmed and about 300 000 disease-related deaths that has affected 188 countries/regions (as of 15^th^ May 2020; 17:30 h CET) (1), the WHO characterized the novel coronavirus COVID-19, first isolated in Wuhan, China (2), as a pandemic on March 11^th^ 2020. Most patients affected were 30–79 years of age (87%) (3) with 14% of cases severe and 5% critical (4). Thus, healthcare practitioners around the world are in need for agility and collaboration toward a common goal (5). Furthermore, one must consider that even a small amount of healthcare resources spent for a non-urgent treatment will be in direct conflict with the greater social good. Additionally, some authorities have ordered requisition of all their registered medical staff, independent of specialisation, so that they can assist wherever needed. This automatically has a direct impact on scheduled therapies. Finally, due to the increasing lockdowns of borders it is difficult, or even impossible, for some nuclear medicine facilities to have access to radioactive iodine (^131^I) or other radioisotopes (especially if only accessible by air).

There have been articles on how radiology and nuclear medicine departments can exercise caution to reduce the risk of an outbreak in their unit (6, 7), but still no advice has been specifically given for nuclear thyroidology. Thyroid nodules are very common, making diagnostic work-up of patients with thyroid nodules a significant activity in many nuclear medicine facilities around the word. Neck ultrasound (with or without fine-needle aspiration cytology (FNA)) is the mainstay in evaluation of thyroid nodules, while radioactive iodine or iodine analogues are well suited for thyroid imaging and radioiodine uptake studies (8). Accordingly, thyroid scintigraphy with either ^99m^Tc-pertechnetate or ^123^I are widely used as an adjunct for the diagnostic evaluation of thyroid nodules (9), while ^131^I-imaging and uptake studies are essential in patients with differentiated thyroid cancer (DTC). Additionally, imaging with specific tracers such as ^99m^Tc-sestamibi (^99m^Tc-MIBI) and ^18^F-fluorodeoxyglucose (^18^FDG) may be useful in selected cases to help in discriminating benign from malignant thyroid nodules (10). Luckily, only less than 10% of thyroid nodules are cancerous (11), and in turn, most thyroid cancers are indolent in nature. Surgery represents the first-line treatment of thyroid cancer (as well as large and symptomatic benign goitres), while risk-adapted ^131^I therapy coupled with whole-body scintigraphy and SPECT/CT is integral in post-operative management of patients affected by DTC. Additional treatments including external radiation therapy, tyrosine-kinase inhibitors and interventional radiology procedures may be required for advanced cancers. The aim of this manuscript is to provide guidance about uniform approaches in nuclear thyroidology in order to reassure patients with thyroid nodules and cancer who are worried about COVID-19 and to avoid the potential negative impact of interruption or postponement of diagnostic and/or therapeutic procedures.

## How will COVID-19 impact on patients with thyroid nodules and cancer?

- The risk to be infected by SARS-CoV-2 and develop critical COVID-19 forms is dictated by demographic characteristics and comorbidities rather than the presence of thyroid diseases, in most cases. Patients with thyroid cancer (TC) who have previously received disease-specific treatment such as surgery (with or without ^131^I administration), as well as the vast majority of those with suspicious thyroid nodule(s) or neck lymphadenopathy, are not considered at higher risk of viral infection including from SARS-CoV-2 (12). The only exception is patients with stage IV disease with severe lung metastatic disease (with also possible radiation-induced lung fibrosis).
- On the other hand, interestingly, significant enhancement of the oncogenic and metastatic potential, through the induction of angiogenesis and changes to the tumor microenvironment, subsequent to viral infection, has been documented for TC (4). However, since COVID-19 is a novel virus, there are no data supporting its oncogenic potential (13).

### (A) How will COVID-19 impact on the diagnosis of thyroid nodules?

- Thyroid nodules are very common, and a substantial majority are benign. The number of thyroid nodules detected with imaging examinations (particularly ultrasonography (US)) has been increasing, but the TC mortality has remained relatively stable (14). While differentiating a neoplastic nodule from a benign one is essential for guiding clinical management, a delay of a few months in diagnosis is unlikely to negatively affect the outcome in most TC patients.
- FNA of most asymptomatic thyroid nodules should be postponed. The clinical picture should be taken into account in addition to the sonographic characteristics of thyroid nodules (using an acceptable scoring such as TIRADS (15)) and guide decisions regarding the need for urgent FNA or surgery (16). Exceptions should include significant symptoms and signs of compression such as rapid enlargement, visible/palpable neck lymphadenopathy, suspicious vocal cord palsy and so forth. These must be promptly recognized and rapidly managed following current guidelines (e.g. with ultrasound and/or cross-sectional imaging and FNA) (17).
- A further exception should include patients with suspicion of medullary thyroid cancer (MTC) (e.g. with family history of MTC), which require prompt evaluation (e.g. serum calcitonin, FNA).
- Concluding, most diagnostic procedures (i.e. thyroid US, scintigraphy, and FNA) can be safely postponed. Notably, request forms and previous reports should be carefully evaluated in order to detect critical cases and proper explanations and information should be provided to patients not requiring urgent evaluation.

### (B) How will COVID-19 impact on therapy for thyroid nodules and thyroid cancer?

Table 1 gives detailed information on the procedures/conditions that should be adapted.

**Table 1:** Procedures that should be adapted during the COVID-19 emergency for thyroid nodules/cancer and radioactive iodine treatment.

Recommendations	Alternatives	Exceptions
Thyroid nodules		
Postponement of scheduled outpatient imaging/functional test (Ultrasonography (US), ^99m^Tc/^123^I/^131^I-scintigraphy with or without %uptake) and fine needle aspiration cytology	Tele-consultation	Patients with significant symptoms, indicating critical events (e.g. pressure to trachea, breathing difficulties) suggesting large goiter should undergo imaging for further assessment (always with precaution and risk/benefit assessment).
	Discuss with referring physician the options of rescheduling and performing as scheduled and come to a consensus	Patients with a history, clinical characteristics and laboratory examinations indicating aggressive thyroid disease e.g. anaplastic, medullary, metastatic or other diseases e.g. lymphoma
		Paediatric patients with non-incidental cervical findings
Postponement of all radioactive iodine (^131^I) therapeutic administration for benign conditions	Rebook and consider a bridging with ATDs until definitive therapy unless contraindicated	Patients contraindicated for ATD’s
Thyroid cancer		
Postponement of diagnostic appointments for all patients with un-/newly diagnosed thyroid cancer and those under suppressive treatment.	Tele- consultation	Patients with a history, clinical characteristics and laboratory examinations indicating aggressive thyroid disease e.g. anaplastic, medullary, metastatic or other diseases e.g. lymphoma
		Paediatric population with non-incidental cervical findings
Postponement of any scheduled outpatient examinations including biochemical and serological labs		High risk patients (and all patients with biochemical incomplete, structural incomplete, or indeterminate response) after careful risk/benefit assessment.
Postponement of any scheduled outpatient imaging/functional test (US, post-surgical scintigraphy with or without % uptake or PET)	Discuss with referring physician rescheduling and performing as scheduled and come to a consensus	Patients with significant symptoms, indicating critical for life events (e.g. pressure to trachea, breathing difficulties) suggesting large goiter (always with precaution and risk/benefit assessment).
Postponement of any scheduled outpatient follow up examinations	Consider serum Tg and TgAbs measurements with or without exogenous TSH stimulation for selected patients.	Patients with local disease possibly infiltrating the trachea or the esophagus, or suspicious liver or bone spread.
Postponement of all non-urgent surgery, even those for cytologically confirmed differentiated thyroid cancer		Patients with large goiter causing regional critical for life events (e.g. pressure to trachea, breathing difficulties) or with rapidly growing thyroid nodules/ cancer
		Paediatric patients with worrisome rate of progression
Postponement of radioactive iodine (^131^I) therapy, either as remnant ablation or as adjuvant treatment (as defined in the Martinique principles (37).		^131^I therapy for which a patient has already begun pre-treatment such as administration of redifferentiating agents or T4 withdrawal
		High risk patients for known disease (as defined in the Martinique principles (37).
Patients on suppressive doses of levothyroxine (i.e. have a TSH target according to their risk profile) should continue their current dose	Dose-adjustment via Tele-consultation	Patients with non-previously existing symptoms of hypo/hyperthyroidism

#### Thyroid cancer

- Most patients with DTC are not at an increased risk to develop critical COVID-19 course, but it should be noted that oncological patients in general suffer from a 3.5 times higher risk of mechanical ventilation, ICU admission or death compared with patients without cancer (18).
- In addition, the minority of TC patients that are receiving multikinase inhibitors (such as lenvatinib or sorafenib) or chemotherapy are at an increased risk of developing adverse events (with their prevalence ranging from 87 to 100%) (19), resulting in a high probability of severe illness from SARS-CoV-2 (12, 13).
- Furthermore, patients who have previously received external beam radiotherapy to the neck may be at increased risk of severe COVID-19 too, requiring individualized and cautious management (20).
- All the aforementioned cases that are at a higher risk of severe/critical infection with COVID-19, including patients with comorbidities, should be shielding and self-isolating. They should systematically follow general principles to help prevent the spread of airway and chest infections, including handwashing and respiratory hygiene, and register for support when needed (21, 22).

## How to manage acutely unwell patients without full nuclear medicine investigation and therapy?

- Rare life-threatening thyroid emergencies, resulting from rapidly increasing neck masses associated with stridor with resultant morbidity, need urgent treatment despite the potential higher risk for critical COVID-19 developing in patients with aggressive cancers (23).
- In such cases a strict cooperation with endocrinologists, endocrine surgeons and other specialists (e.g. medical/radiation oncologists) is advised in order to provide an appropriate and timely treatment (24, 25).
- Patients requiring surgical intervention include those with evidence of aerodigestive tract compromise/invasion; recurrent laryngeal nerve palsy due to malignancy; locoregional metastasis; large, compressive tumours; clinical concern for example rapid growth, poorly differentiated.
- Early diagnosis and surgery of MTC significantly improves outcomes (26). MTC should be managed as clinically appropriate, i.e. by adhering as closely as possible to usual practice. MEN2 - prophylactic surgery in paediatric MEN2 patients, with a genetic profile suggesting a statistically high risk of developing malignancy, should not have surgery deferred wherever possible. If surgery is deferred, monitor calcitonin levels and ultrasound findings carefully and upgrade to urgent if indicated (27).
- Anaplastic thyroid cancer (ATC) is one of the few occasions when thyroid surgery should be performed on an urgent basis. Patients typically present with a rapidly enlarging thyroid mass that is associated with compressive symptoms, such as dysphagia and dyspnoea (16).

## How should current patients with thyroid nodule/cancer be advised about risk? Who still needs to be seen and why? What/who can be converted safely to remote review?

- In general, nuclear thyroidology procedures are not urgent for example due to the indolent natural history of most of DTCs (28). There is little evidence that early detection and treatment of DTC significantly alters disease outcomes, as the imaging-driven rise of incidence has not negatively impacted the excellent overall survival (14).
- Current patients with thyroid nodule(s) or TC, and those scheduled for radioactive iodine treatment, merit a detailed consultation on the particularity of the situation and the reasons that have led to the variation of the scheduled procedure. Most importantly, the patients should understand that the decisions made were based on thoughtful assessment of risks and benefits and were not a product of reckless judgment or fear. Extensive time should be planned for such remote consultations and ideally the patient should be notified a priori in order to be better prepared, so that they can, for example, write down questions needing clarification. Furthermore, specific online frequently asked questions (FAQ) on issues of interest could be offered from each institution to inform their patients for example on the risk of postponing a surgery of DTC. A plethora of online sources of information from different societies/groups specifically relating to COVID-19 and thyroid nodules and cancer are currently available, where patients could be referred to (29, 30, 31, 32, 33, 34). The key precondition to ensure safe and effective diagnostic and therapeutic management of TC patients, without jeopardizing the implications of a possible COVID-19 infection, is an individualized approach and cautious risk/benefit assessment.
- After an individual risk-benefit analysis, therapy with ^131^I may proceed for selective cases, despite the threat of COVID-19 infection (as stated in Table 1). All remaining patients can be safely reviewed remotely based on risk, in predefined time intervals, with an ‘open line’ for the patient to report abnormalities.

## How should nuclear thyroidology services be remodelled in acute crisis?

- The challenge for the nuclear medicine physician lies upon weighing up the risk of a delay in diagnosis and treatment of thyroid diseases against the patient’s risk to be affected by COVID-19, especially in higher risk groups. Of course, thyroid nuclear imaging and/or therapy with ^131^I must be guaranteed in selective cases, despite the threat of SARS-CoV-2 infection and COVID-19. Liang *et al.* were the first to publish suggestions for nuclear medicine departments (both for patients and staff) (7). Among other suggestions, triage both for patients and staff members at the hospital entrance is of highest importance in order to timely identify symptomatic patients (e.g. fever, tiredness, dry cough). Furthermore, camera gantries and all other surfaces coming in contact with patients should be wiped with disinfectant regularly and after every contact. Staff seeing patients should be minimized and a contingency and business continuity plan should be developed if one of the staff members becomes sick with COVID-19. Additionally, staff members with risk factors should be given extra care and instructed to stay at, and if possible, work from home.
- Should thyroid FNA be undertaken, it is advised that patients are tested for COVID-19 by undergoing the available nucleic acid test, in additional to observing the guidance of utilising personal protective equipment for both the patient and staff (16).
- In addition to these suggestions (7), treating physicians should bear in mind the possibility that a treated patient could potentially be infected before, during or after hospitalisation. Thus, the patient may require further specialized/acute care within or outside radioprotected areas (i.e. ICU) after discharge. Therefore, and to the extent possible, discharge rules should be applied more strictly and according to national rules (i.e. the discharge rules for the allowed residual radioactivity in each country should be strictly followed and, if meaningful, patients should be discharged with even lower levels), so that other healthcare practitioners and the general public remain protected in such a scenario.
- For non-urgent cases virtual health care is an excellent option for patient communications during the pandemic and can significantly help to minimize the time of face-to-face contacts, thus slowing transmission and flattening the curve. Providing distant digital care does not have to be complicated.

## What online resources are available; what will be necessary?

- There are so many ways to monitor people’s health that we are not currently practicing at any scale, in large part due to regulatory barriers. As stated from experts, virtual health care is inexpensive and practical, but it will never be the same as a physical examination. However, with COVID-19 this is a trade-off we must accept as the risk–benefit ratio for virtual health care has massively shifted (35). Most patients and doctors are able to use modern media such as computers, tablets and smartphones, to connect and participate in video/ audioconferencing (e.g. using popular apps that allow for video chats such as WhatsApp, Apple FaceTime, Facebook Messenger etc.) and exchange information. Although mobile phone use now is globally ubiquitous, patient/practitioner populations without access/basic knowledge to such means (e.g. elderly) should be encouraged to learn them.
- Justifiable regulatory barriers should however be followed or revised. While some authorities are just beginning to consider regulating virtual health care, others are already in the process of updating existing rules during the pandemic. Following China’s example, on March 17^th^, the U.S. Health Insurance Portability and Accountability Act waived potential penalties for violations against health care providers that serve patients through everyday communications technologies during the COVID-19 emergency, in order to provide maximum flexibility to respond to the pandemic (35). In Europe, although nearly all European Data Protection Authorities are in agreement that although ‘data protection laws do not stand in the way of the provision of healthcare and the management of public health issues, important considerations with respect to handling of personal data and particularly, sensitive medical data’, should be taken under advisement. The management of the COVID-19 crisis also does not excuse data controllers from the applicability of other GDPR-related fundamentals and efforts that are being made (36).
- At the same time, it is crucial that national health care systems recognize the importance of this approach and provide motives and reimbursement options for healthcare practitioners to increase the likelihood that virtual health care will be embraced adequately. China’s virtual healthcare transformation was unleashed when the country’s national health insurance agency agreed to reimburse virtual care consultations (35).
- Furthermore, if the restrictions apply over a longer period, health care systems should expand their digital functionality and build their own, or encourage the use of digital platforms with additional functions such as electronic scheduling, analytics and reports, image and file uploads and e-prescribing.

## What might be the longer-term consequence for service provision?

- Backlog of patients who have had their surgery and treatment postponed should be prioritized based on risk (e.g. presentation status, histological subtype, ultrasound features etc.). Furthermore, low ^131^I activities could be administrated whenever possible in different hospitals, so that possible allowed thresholds of ^131^I usage and/or waste disposal etc. will not be exceeded.
- SARS-CoV-2 exposes the health system vulnerability. The knock-on costs will be immense, and, after the shock, we must recover quickly. This is crucial for institutions that are cancelling (correctly, at this time) nonurgent procedures to spare resources for costly COVID-19 patients and are more likely to face a significant financial impact of the pandemic, long after it recedes. These knock-on effects will be more profound for smaller health systems that cannot compensate adequately.
- Thus, governments should fund the health care providers that are on the front lines of this pandemic based on the financial losses registered and give motives for clinicians (e.g. reimbursing telemedicine) for a healthy and qualitative restart of the health systems. On the other hand, the current crisis provides an excellent opportunity to retain the better parts of models currently forcibly being tested, such as virtual health care.
- The sacrifice – just like other cases – will most likely be research, which will have a negative impact on health care systems over the longer term. Maximum care should be taken to ensure that medical research will not unduly suffer from the fall-out of the current COVID-19 pandemic, as it is only with ongoing research that we, as physicians, can continue to improve healthcare and quality of life of patients

## Disclaimer

Due to the emerging nature of the COVID-19 crisis this document is not based on extensive systematic review or meta-analysis, but on rapid expert consensus. The document should be considered as guidance only; it is not intended to determine an absolute standard of medical care. Healthcare staff need to consider individual circumstances when devising the management plan for a specific patient.

## Declaration of interest

The authors declare that there is no conflict of interest that could be perceived as prejudicing the impartiality of this guidance.

## Funding

This work did not receive any specific grant from any funding agency in the public, commercial, or not-for-profit sector.
